# High prevalence of symptoms among Brazilian subjects with antibodies against SARS-CoV-2

**DOI:** 10.1038/s41598-021-92775-y

**Published:** 2021-06-24

**Authors:** Ana M. B. Menezes, Cesar G. Victora, Fernando P. Hartwig, Mariângela F. Silveira, Bernardo L. Horta, Aluísio J. D. Barros, Marilia A. Mesenburg, Fernando C. Wehrmeister, Lúcia C. Pellanda, Odir A. Dellagostin, Cláudio J. Struchiner, Marcelo N. Burattini, Fernando C. Barros, Pedro C. Hallal

**Affiliations:** 1 Universidade Federal de Pelotas, Pelotas, Brazil; 2 Fundação Universidade Federal de Ciências de Saúde de Porto Alegre, Porto Alegre, Brazil; 3 Fundação Getúlio Vargas, Rio de Janeiro, Brazil; 4 Universidade de São Paulo, São Paulo, Brazil

**Keywords:** Viral infection, Fatigue, Fever, Respiratory signs and symptoms

## Abstract

Since the beginning of the pandemic of COVID-19, there has been a widespread assumption that most infected persons are asymptomatic. Using data from the recent wave of the EPICOVID19 study, a nationwide household-based survey including 133 cities from all states of Brazil, we estimated the proportion of people with and without antibodies for SARS-CoV-2 who were asymptomatic, which symptoms were most frequently reported, number of symptoms and the association with socio-demographic characteristics. We tested 33,205 subjects using a rapid antibody test previously validated. Information was collected before participants received the test result. Out of 849 (2.7%) participants positive for SARS-CoV-2 antibodies, only 12.1% (95% CI 10.1–14.5) reported no symptoms, compared to 42.2% (95% CI 41.7–42.8) among those negative. The largest difference between the two groups was observed for changes in smell/taste (56.5% versus 9.1%, a 6.2-fold difference). Changes in smell/taste, fever and body aches were most likely to predict positive tests as suggested by recursive partitioning tree analysis. Among individuals without any of these three symptoms, only 0.8% tested positive, compared to 18.3% of those with both fever and changes in smell or taste. Most subjects with antibodies against SARS-CoV-2 are symptomatic, even though most present only mild symptoms.

## Introduction

Since the beginning of the pandemic of COVID-19, there is a widespread notion that most people infected by SARS-CoV-2 are asymptomatic, following an early article from China stating that 86% of those infected did not report any symptoms^1^. More recently, several clinical studies became available, showing that the prevalence of asymptomatic infected individuals ranges from 4 to 75%^2–6^. These discrepancies might be explained by the use of different lists of symptoms, different recall periods, as well as different populations. Population-based studies are particularly relevant for studying SARS-CoV-2 symptoms, because asymptomatic patients or those with mild symptoms may be identified at home, rather than in health service-based studies.

Using data from the most recent wave of the EPICOVID19 study, a nationwide household-based survey including 133 cities from all states of Brazil^7^, we estimate the proportion of people with and without antibodies for SARS-CoV-2 who were asymptomatic. We investigated which symptoms were most frequently reported, how many symptoms were reported by each subject, and the associations between symptoms and sociodemographic characteristics. We also performed conditional inference tree analyses using binary recursive partitioning to identify which combinations of symptoms were most likely to predict positive test results.

## Methods

EPICOVID19 is a nationwide seroprevalence survey conducted in sentinel cities in 26 Brazilian states and the Federal District. The Brazilian Institute of Geography and Statistics (IBGE) divides the country into 133 intermediate regions, and the most populous municipality in each region was included in the sample. So far, the study has entailed three waves of data collection (May 14–21, June 4–7, and June 21–24). Subjects were told that the objective of the study was to identify the number of people infected by SARS-CoV-2.

Here we report on findings from the third wave of data collection which included a detailed investigation of symptoms.

A multi-stage probabilistic sample was adopted, with 25 census tracts selected in each one of the 133 sentinel cities, with probability proportionate to size. In each sampled tract, 10 households were systematically selected, totaling 250 households per municipality. All household residents were listed, and age and sex recorded on a list. One individual was then randomly selected as the respondent for that household. Then, a finger prick blood sample was obtained and a questionnaire applied. If the selected subject did not accept to participate, a second resident was randomly chosen. In case of another refusal, the interviewers moved to the next household to the right of the one that had been originally selected; different households were selected in each wave of the study. The total planned sample size was 33,250 individuals.

The WONDFO SARS-CoV-2 Antibody Test (Wondfo Biotech Co., Guangzhou, China) was used for the detection of antibodies for SARS-CoV-2 (https://en.wondfo.com.cn/product/wondfo-sars-cov-2-antibody-test-lateral-flow-method-2/); this rapid point-of-care test is based on the principle of immune assay of lateral flow and detects IgG/IgM antibodies against SARS-CoV-2. The presence of antibodies is detected by two drops of blood from a pinprick sample; after the introduction of the blood sample, valid tests are identified by a positive control line in the kit’s window; if this control line is not visible, the test is considered inconclusive. A second line also appears in the window if SARS-CoV-2-reactive antibodies are present; in the absence of antibodies, this line is not visible. This rapid test underwent independent validation studies; by pooling the results from the four validation studies, weighted by sample sizes, sensitivity was estimated at 84.8% (95% CI 81.4%;87.8%) and specificity at 99.95% (95% CI 97.8%;99.7%)^8–10^.

Field workers used tablets to record the full interviews, registered all answers, and photographed the test results. All positive or inconclusive tests were read by a second observer, as well as 20% of the negative tests. Subjects were asked about presence (yes/no) of 11 symptoms since March 2020, when the first cases were reported in Brazil: fever, sore throat, cough, difficulty breathing, palpitation, changes in smell or taste, diarrhea, vomiting, body aches (the question was phrased as “aches in the whole body”), shivering and headache. Subjects were classified as “asymptomatic” if they answered “no” for all symptoms.

Sociodemographic variables were also investigated: sex, age in years, schooling (last year completed/grade; recoded as primary or less; secondary; university or higher), self-reported skin color, and household assets. The official Brazilian classification of ethnicity recognizes five groups, based on the question: “What is your race or color?” The five response options are “white”, “brown” (“pardo” in Portuguese), “black”, “yellow” and “indigenous”. Interviewers were instructed to check the “yellow” option when the respondent mentions being of Asian descent, and “indigenous” when any of the multiple first nations were mentioned^11^.

The wealth index was created based on a list of assets and goods (computer or laptop, internet access, color television, air conditioning equipment, number of vehicles, cable TV, number of bathrooms and number of bedrooms), through a principal component analysis. The first component was extracted and the total sample divided into quintiles weighted by municipality urban population; the first quintile represent the 20% poorest individuals, and the fifth quintile represents the wealthiest 20% in the sample^12^. For the schooling analysis, subjects under 25 years were excluded as they could still be attending school.

Interviewers were tested prior to the field work and only those found to be negative for the virus could participate in the study. Biological safety measures were taken to protect the health of the field workers and individual protection equipment was discarded after visiting each household. Ethical approval was provided by the Brazilian’s National Ethics Committee (process number: 30721520.7.1001.5313). Study participants were informed about the objectives of the study, possible risks and advantages; for subjects under 18, consent was obtained from a parent and/or legal guardian. Blood collection took place after obtaining written informed consent from participants or their legal guardians. Individuals testing positive were referred to the statewide COVID-19 surveillance system. In case of a positive rapid test by the respondent, all other residents of the household were also tested for antibodies. All methods were performed in accordance with the relevant guidelines and regulations.

The prevalence of each of the 11 symptoms was calculated separately for individuals who tested positive and those with negative results. Means and standard errors (SE) were estimated for the variable on number of symptoms. T-student tests or ANOVA were applied, according to the type of exposure variable. Prevalence ratio and 95% confidence interval (95% CI) were calculated for each symptom, by dividing the frequency of each symptom in positive and negative subjects. Chi-squared test for heterogeneity or linear trend were calculated, according to the type of variable studied. Subjects with previous diagnosis of COVID-19 (n = 242) and missing information on symptoms (n = 1104) were excluded from the analysis.

We also performed conditional inference tree analyses using binary recursive partitioning, accounting for multiple testing^13^. The objective of these analyses was to identify which combinations of the 11 symptoms were most likely to predict positive test results.

Analyses were performed using the software Stata version 14.1 (StataCorp, College Station, TX, USA) and conditional inference tree analyses were performed using R 3.6.1 (https://www.r-project.org/). Data will become publicly available 30 days after completion of the fieldwork at http://www.epicovid19brasil.org/.

## Results

Of the target sample size comprising 33,250 individuals, we were able to include 33,205 (99.9%) participants in the study. To achieve this number, a total of 59,724 houses were contacted, with 19.8% of refusals and 24.6% of houses being empty at the time of the visit. Of the 31,869 participants included (after excluding for missing on symptoms and previous COVI-19 diagnosis), 849 subjects (2.7%) tested positive for SARS-CoV-2 antibodies. Test results were only disclosed after the interview on symptoms had been completed. Table 1 shows the distribution of the sample and the prevalence of positive antibody tests according to sociodemographic characteristics.

**Table 1:** Distribution of the study sample and prevalence of positive antibodies for SARS-CoV-2, according to sociodemographic characteristics and region.

	Sample distribution		% of positive tests
	Number	%	
**Region**			
Northeast	9982	31.3%	6.2%
North	5180	16.3%	4.1%
Central-West	3603	11.3%	0.9%
Southeast	8021	25.1%	0.4%
South	5083	16.0%	0.7%
**Sex**			
Female	18,646	58.5%	2.7%
Male	13,223	41.5%	2.6%
**Age (years)**			
0–4	637	2.0%	4.6%
5–9	862	2.7%	2.7%
10–19	2789	8.8%	1.9%
20–29	4965	15.6%	2.4%
30–39	4999	15.7%	3.0%
40–49	5078	15.9%	2.8%
50–59	5032	15.8%	3.0%
60–69	4234	13.3%	2.6%
70 +	3273	10.3%	2.1%
**Color/ethnicity**			
White	11,442	36.7%	1.3%
Brown	14,131	45.4%	3.5%
Black	4264	13.7%	2.9%
Asian	897	2.9%	3.0%
Indigenous	429	1.4%	6.3%
**Schooling**			
Primary or less	11,417	39.3%	2.8%
Secondary	11,363	39.1%	2.8%
University or higher	6275	21.6%	1.4%
**Wealth quintiles**			
Poorest	7668	24.1%	3.3%
2nd	5809	18.2%	3.3%
3rd	6334	19.9%	2.8%
4th	6214	19.5%	2.4%
Richest	5844	18.3%	1.4%

The EPICOVID19 study, third wave.

Each of the 11 symptoms investigated were significantly (P < 0.01) more likely to be reported by those testing positive as compared to those testing negative (Table 2). The most frequently reported symptoms among positive cases were headaches (58.0%), changes in smell or taste (56.5%), fever (52.1%), cough (47.7%) and body aches (44.1%). Table 2 also presents the prevalence ratios for each symptom and the 95% CI according to SARS-CoV-2 antibodies. The largest ratios between positive and negative subjects were observed for changes in smell or taste (6.2-fold), fever (4.3-fold), shivering (3.3-fold) and body aches (2.8-fold). The sensitivity and specificity for positive test results, for each symptom, are presented in Supplementary Table 1. The two symptoms with sensitivity above 50% and specificity above 85% were changes in smell or taste, followed by fever.

**Table 2:** Prevalence of symptoms among subjects with positive and negative antibody tests for SARS-CoV-2, and prevalence ratios.

Symptom	Prevalence		Prevalence ratio	95% CI	
	Positive	Negative		Lower bound	Upper bound
Headaches	58.0	35.5	1.6	1.5	1.8
Changes in smell or taste	56.5	9.1	6.2	5.6	6.8
Fever	52.1	12.2	4.3	3.9	4.7
Cough	47.7	22.2	2.1	1.9	2.4
Body aches	44.1	15.7	2.8	2.5	3.1
Sore throat	33.8	16.6	2.0	1.8	2.3
Diarrhea	25.6	11.7	2.2	1.9	2.5
Difficulty breathing	23.1	9.4	2.5	2.1	2.8
Shivering	20.5	6.1	3.3	2.9	3.9
Palpitation	20.0	10.5	1.9	1.6	2.2
Vomiting	9.5	4.0	2.4	1.9	3.0

The EPICOVID19 study, third wave.

**Figure 1:**
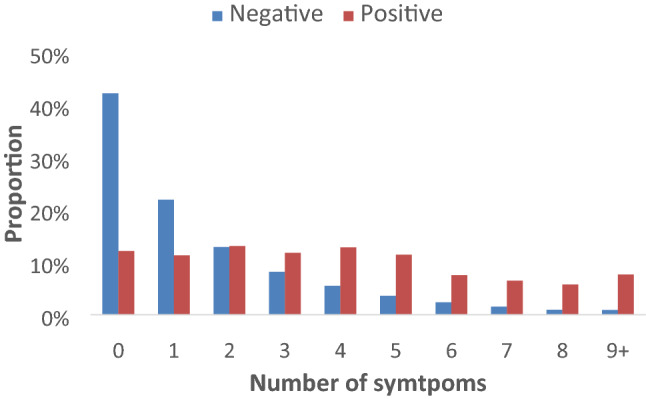
Distribution of the number of symptoms in individuals positive and negative for antibodies for SARS-CoV 2. The EPICOVID19 study, third wave.

Of the 849 participants who tested positive for SARS-CoV-2 antibodies, only 12.1% (95% CI 10.1–14.5) reported none of the 11 symptoms and were therefore classified as asymptomatic, against 42.2% (95% CI 41.7–42.8) among those who tested negative (Fig. 1). The mean (SE) number of symptoms for those who tested positive or negative were 3.91 (0.10) and 1.53 (0.01), respectively. Among those who tested positive, 63.5% had three or more symptoms, compared to 23.0% among those who tested negative.

In Table 3 we present the mean number of symptoms and the prevalence of asymptomatic subjects, stratified by antibodies test status (positive or negative) according to sociodemographic characteristics. Subjects who tested negative tended to have lower frequency of symptoms than positive subjects, within all categories of sociodemographic variables and patterns of symptoms tended to be similar for both groups of positive and negative subjects.

**Table 3:** Percent asymptomatic and mean number of symptoms in subjects positive and negative for antibodies against SARS-CoV-2, according to sociodemographic characteristics.

	Positive individuals (N = 849)				Negative individuals (N = 31,867)			
	% asymptomatic	Mean number of symptoms	95% CI		% asymptomatic	Mean number of symptoms	95% CI	
**Sex**								
Male	13.5	3.6	3.3	3.9	48.8	1.2	1.2	1.3
Female	11.1	4.1	3.9	4.4	37.5	1.7	1.7	1.8
**Skin colour**								
White	12.4	3.7	3.2	4.1	44.3	1.4	1.3	1.4
Brown	10.2	4.1	3.9	4.4	40.7	1.7	1.6	1.7
Black	15.6	3.7	3.2	4.2	42.6	1.5	1.5	1.6
Yellow	14.8	3.7	2.6	4.7	37.4	1.7	1.5	1.8
Indigenous	22.2	3.3	2.2	4.3	38.1	1.7	1.5	2.0
**Age**								
1 to 4	27.6	2.4	1.5	3.3	50.3	1.1	0.9	1.2
5 to 9	21.7	2.4	1.7	3.2	52.3	1.1	1.0	1.2
10 to 19	5.7	3.1	2.5	3.7	36.9	1.6	1.5	1.7
20 to 29	8.3	4.1	3.6	4.6	33.1	1.9	1.8	2.0
30 to 39	6.7	4.9	4.5	5.4	38.3	1.8	1.7	1.9
40 to 49	13.9	4.3	3.8	4.7	41.3	1.7	1.6	1.7
50 to 59	15.0	3.8	3.4	4.3	44.9	1.4	1.4	1.5
60 to 69	12.0	3.5	3.0	4.0	50.5	1.2	1.1	1.2
70 or more	15.9	3.2	2.6	3.8	48.8	1.1	1.1	1.2
**Wealth index**								
Poorest	13.5	3.8	3.5	4.2	43.4	1.6	1.5	1.6
2nd	13.2	3.9	3.5	4.3	41.2	1.6	1.5	1.7
3rd	11.4	4.0	3.6	4.5	42.2	1.6	1.5	1.6
4th	13.2	3.8	3.4	4.3	41.0	1.5	1.5	1.6
Richest	5.0	4.1	3.6	4.7	43.0	1.4	1.3	1.4
**Schooling**								
Primary or less	14.3	3.6	3.3	3.9	45.0	1.4	1.4	1.5
Secondary	8.9	4.3	4.0	4.6	39.8	1.7	1.6	1.7
University or higher	7.8	4.4	3.9	5.0	38.7	1.6	1.6	1.7

The EPICOVID-19 study, third wave.

**Figure 2:**
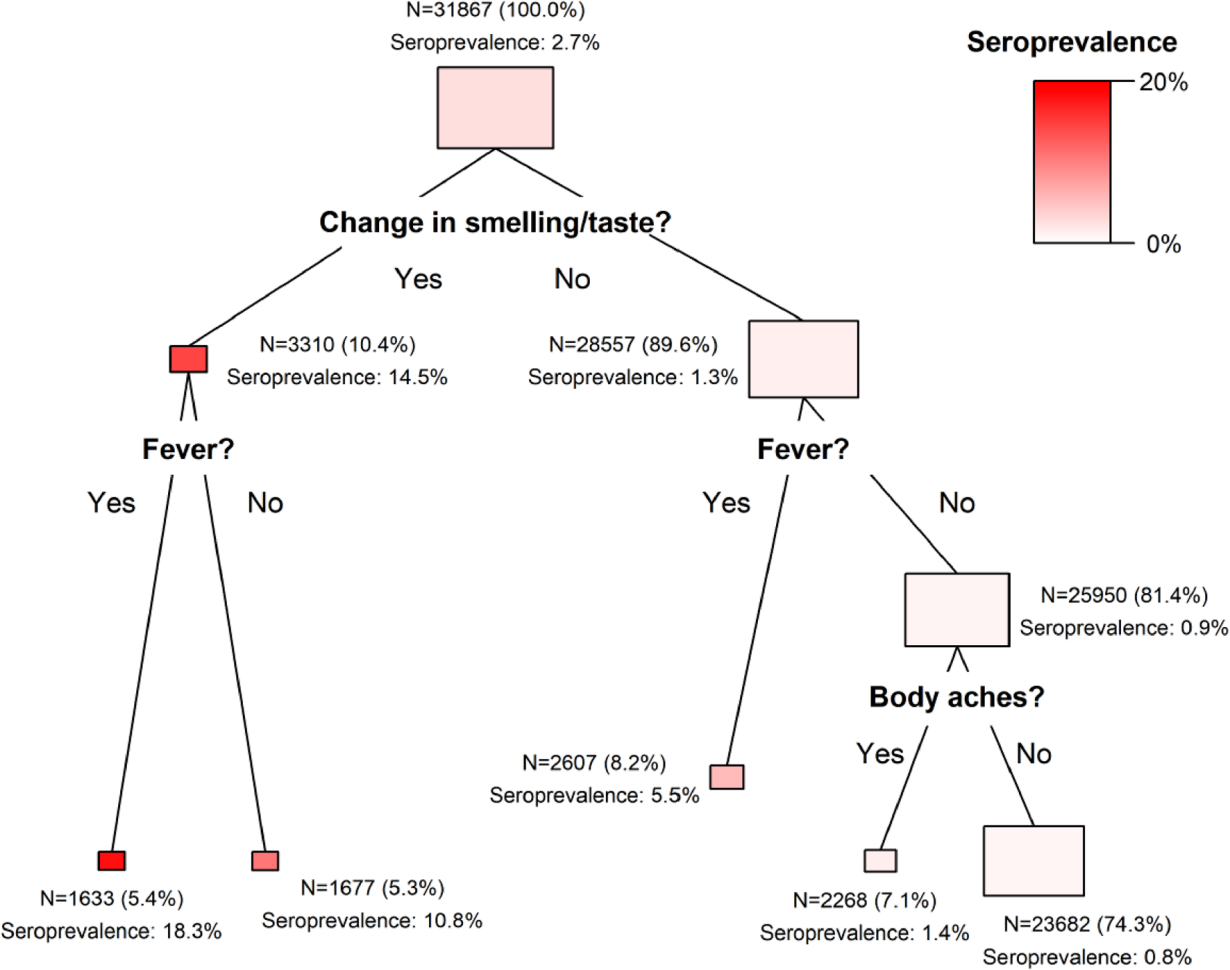
Conditional inference tree of the association between symptoms (predictors) and seroprevalence for SARS-CoV 2. The EPICOVID19 study, third wave. The area of the rectangles corresponds to the proportion of the population contained in each node.

Figure 2 displays the results of the conditional inference tree analysis. Out of the 11 symptoms, this analysis selected three: changes in smell or taste, fever and body aches. Given the low overall seroprevalence, in all terminal nodes the prevalence was lower than 20%. Notably, the two thirds of the total sample who reported none of the three symptoms presented a markedly low seroprevalence of 0.8%, compared to 18.3% among those presenting fever, body aches and changes in smell or taste.

When an individual tested positive, we also tested other family members, but we did not record symptoms among them. Of the 90 positive subjects with at least one positive family member, 6.7% were asymptomatic, compared to 13.0% asymptomatic among 747 positive subjects without any positive family members. Lastly, we verified whether antibody prevalence levels in cities were associated with the frequency of symptoms among positive subjects, and found no such association (Supplementary Fig. 1).

## Discussion

In the first two waves of the EPICOVID19 nationwide survey, we identified that, contrary to what is often reported, most subjects with antibodies were symptomatic. However, symptoms had only been assessed for those with positive tests, and the information was collected after the individual had learned about the test result. We addressed the possibility of bias by asking all participants, regardless of the test result, in the third wave. The question on symptoms covered the four-month period since the first COVID-19 cases were reported in the country. The questionnaire was applied before the test result was known, so that respondents were blind to their serological status, and this allowed us to compare symptoms among those testing positive and those testing negative. Subjects with a previous diagnosis of COVID-19 as well as those with missing information for symptoms (0.73% of the whole sample) were excluded from the analyses in order to ensure that the respondents were not aware of their condition. Questions regarding symptoms were only applied to index subjects, and not to other members of the family who tested positive for SARS-CoV-2.

The above results from the third wave of the study confirmed a high prevalence of symptoms using a 4-month recall period; only 12.1% positive subjects were asymptomatic, compared to 42.2% of those without antibodies. Inclusion in our analyses of individuals who tested negative was useful for identifying which symptoms were most strongly associated with the presence of antibodies. For example, headaches were the most common symptom affecting 58.0% of those positive, but were also reported by 35.5% of those who tested negative, a prevalence ratio of only 1.6. In contrast, changes in smell or taste affected 56.5% among those who tested positive and 9.1% in the negative ones, respectively. This symptom provided the best discrimination, with a prevalence ratio of 6.2. Recent studies have shown that when SARS-CoV-2 enters the nasal and oral epithelium through the angiotensin-converting enzyme 2 (ACE2) and transmembrane serine protease 2 (TMPRSS2), it may cause damages to olfactory and gustatory receptor cells resulting in anosmia or ageusia^14,15^.

Overall, symptoms were more frequent among females than males, in subjects aged 30–29 years and in those with higher education. Children and adolescents were substantially less likely to report symptoms than adults, which is compatible with the lower infection-fatality rates observed in these age groups^16^. In contrast, prevalence of symptoms fell with age from 30 to over 70 years, which does not reflect the age pattern in infection-fatality and case-fatality^17^. The difference in reported symptoms between women and men is also at odds with the higher case-fatality among males^18^. Although we did not record severity of symptoms, the number of symptoms reported is likely to indicate more severe cases.

Comparison of our findings on the prevalence of symptoms with the literature are affected by the settings in which studies were done, by the phase of infection, the duration of recall, and by the ways in which symptoms were recorded, as well as whether or not the subjects were aware or suspicious of being infected. The prevalence for asymptomatic subjects in the literature ranges from 4 to 75%^2–6,19,20^, whereas in our study it was 12.1%. We identified five published reviews that provided pooled prevalence estimates for symptoms^4,5,21–23^ among individuals who tested positive in health facilities. We found lower prevalence (52.1%) for fever (pooled prevalence ranging from 78.4% to 92.8%) and cough (47.7% versus pooled prevalence ranging from 58.3% to 72.2%). Our estimates for body aches (44.1%) and difficulty breathing (23.1%) were within the ranges reported in the studies (29.4% to 51.0%, and 20.6% to 45.6%, respectively). Lastly, prevalence of headache in our study (58%) was considerably higher than in the reviews (8.0% to 14.0%). One may assume the prevalence ranges of symptoms based on individuals who sought care in medical facilities would tend to be higher than in our population-based survey, but this was not the case, except for fever or cough. Notably, changes in smell or taste was not investigated in these review papers. A review of the literature, using changes in smell or taste or anosmia/ ageusia as a search keywords, identified a few articles with prevalence varying from 5.1 to 85.6%^14,24–26^, whereas we found 56.5%.

Besides the aforementioned symptoms, some studies have hypothesized that the angiotensin-converting enzyme 2 receptor (ACE2) is also expressed in the mucosa of the gastrointestinal (GI) tract and play lead to GI manifestations^27^. The pooled prevalence of GI symptoms has ranged in the literature from 7.4 to 12.5% for diarrhea (against 25.6% in our study), and 4.6–10.2% for nausea and/or vomiting (compared to 9.5% in our study)^27–29^.

It is likely that the information on symptoms from population-based studies, such as the one from Spain^30^, would be comparable to our study; however, the recall time in that study was two weeks, compared to up to four months in our survey. In this study, the only symptom specifically reported was anosmia, that was present around 27% of positive subjects, in the three waves.

The decision tree analyses were useful for identifying a subgroup of individuals who presented both fever and changes in smell or taste, among whom seroprevalence was 18.3%, compared to only 0.8% among subjects that did not present these two symptoms, nor presented body aches.

It is clear from the literature that no single symptom correlates perfectly with SARS-CoV-2 infection, thus raising the possibility that the use of multiple symptoms might be appropriate for screening purposes. However, the literature on this topic is still scarce^31^. A study using app-based self-reported data in the United States and in the United Kingdom identified that changes in smell or taste is the single symptom most strongly correlated with infection and, using stepwise logistic regression, identified a prediction model that also includes fatigue, persistent cough and loss of appetite^32^. We also identified changes in smell or taste as the single most predictive symptom, but the two additional symptoms prioritized in the conditional inference tree analysis were fever and body aches. Given that the symptoms are partially correlated to one another, it is possible that models including different symptoms yield similar predictions, and would therefore be of similar practical use. Another app-based study including mostly individuals in the United Kingdom identified that, collectively, symptoms improve predicting prognosis^33^. This indicates that symptoms may be used not only for screening, but also for patient monitoring and planning health service needs.

Our study has limitations. Recall bias is a concern, particularly by using a 4-month recall period, but the alternative—as in the Spanish survey—was to ask for symptoms in a shorter, more recent period and potentially misclassifying individuals who had the disease in the past, and for whom antibodies remained detectable. In order to evaluate the likelihood of recall bias, we excluded the 242 participants who had a diagnosis of COVID-19 prior to the interview. Another limitation is the growing evidence that antibody levels decrease rapidly over time, for example by 14% in the same subjects in the Spanish study^30^, by 30% in a Brazilian cohort in the Amazon state in Brazil^34^ and in our own (unpublished) analyses comparing the first and third waves of the survey in cities with high initial prevalence. This would lead some individuals who had the disease to test negative, and yet report symptoms that occurred at the time of the episode. This type of bias would reduce the difference in reported symptoms among subjects who tested positive and negative. Although the possibility of such bias, at the time of the early rounds of the EPICOVID-19 study, the only rapid test available in large amounts in the country was the Wondfo lateral-flow test, which had been donated to the Ministry of Health in Brazil.

It is possible that individuals who tested positive represent more severe cases and therefore reported a larger number of symptoms. This type of bias is likely to affect studies based on antibody testing as well as studies with clinical illness who sought health services.

We should also to point out that other infectious diseases, such as dengue, chikungunya, zika, and malaria continued to affect the Brazilian population in endemic areas, co-existing with the COVID-19 pandemic. Such outbreaks may result in symptoms that overlap with those due to the pandemic^35^.

The households in each wave of the study were distinct, aiming to avoid bias resulting from repeated interview in the same households.

Positive aspects of our study, on the other hand, included the population basis over an area of 8.5 million square km, the large sample size, collection of symptoms in positive and negative cases, and blinding of respondents as test results were only disclosed after the clinical history was collected.

In summary, our analyses show that most infected SARS-CoV-2 subjects in Brazil are symptomatic, even though most subjects present only mild symptoms. Our findings can be used to implement surveillance systems in Brazil, which would help identify cases early and guide testing procedures.

## Supplementary Information

Supplementary Information.

## References

[1] Li R (2020). Substantial undocumented infection facilitates the rapid dissemination of novel coronavirus (SARS-CoV-2). Science.

[2] Zhou X, Li Y, Li T, Zhang W (2020). Follow-up of asymptomatic patients with SARS-CoV-2 infection. Clin. Microbiol. Infect..

[3] Day M (2020). Covid-19: Identifying and isolating asymptomatic people helped eliminate virus in Italian village. BMJ.

[4] Zhu J (2020). Clinicopathological characteristics of 8697 patients with COVID-19 in China: A meta-analysis. Fam. Med. Community Health.

[5] Fu L (2020). Clinical characteristics of coronavirus disease 2019 (COVID-19) in China: A systematic review and meta-analysis. J. Infect..

[6] Kronbichler A (2020). Asymptomatic patients as a source of COVID-19 infections: A systematic review and meta-analysis. Int. J. Infect. Dis..

[7] Hallal, P. *et al.* EPICOVID19 protocol: repeated serological surveys on SARS-CoV-2 antibodies in Brazil. Ahead of print. (Accessed 31 July 2020); http://www.cienciaesaudecoletiva.com.br/artigos/epicovid19-protocol-repeated-serological-surveys-on-sars-cov2-antibodies-in-brazil/17691 (2020). 10.1590/1413-81232020259.25532020 32876244

[8] Whitman, J. D. *et al. *Evaluation of SARS-CoV-2 serology assays reveals a range of test performance. *Nat Biotechnol*. **38**(10), 1174–1183 (2020). 10.1038/s41587-020-0659-0 PMC7740072 32855547

[9] Horta BL (2015). Cohort profile update: The 1982 Pelotas (Brazil) Birth Cohort Study. Int. J. Epidemiol..

[10] Pellanda, L. C. *et al.* Sensitivity and specificity of a rapid test for assessment of exposure to SARS-CoV-2 in a community-based setting in Brazil, Preprint. (Accessed 31 July 2020); https://www.medrxiv.org/content/, 10.1101/2020.05.06.20093476v1 (2020).

[11] Petruccelli, J. L. & Saboia, A. L. *Características étnico-raciais da população: classificações e identidades*. Instituto Brasileiro de Geografia e Estatística-IBGE, Rio de Janeiro, Brasil (2013). 208pp. (Accessed 31 July 2020); https://biblioteca.ibge.gov.br/visualizacao/livros/liv63405.pdf.

[12] Rutstein, S. O. *The DHS wealth index: approaches for rural and urban areas*. Macro International Incorporated, Calverton, Maryland, USA (2008). (Accessed 20 July 2020); https://dhsprogram.com/publications/publication-wp60-working-papers.cfm.

[13] Hothorn T, Hornik K, Zeileis A (2006). Unbiased recursive partitioning: A conditional inference framework. J. Comput. Graph. Stat..

[14] Hoang, M. P. *et al.* Olfactory and gustatory dysfunctions in COVID-19 patients: A systematic review and meta-analysis. *Asian Pac. J. Allergy Immunol*. **38**(3), 162–169 (2020). 10.12932/AP-210520-0853 32563232

[15] Butowt R, Bilinska K (2020). SARS-CoV-2: Olfaction, brain Infection, and the urgent need for clinical samples allowing earlier virus detection. ACS Chem. Neurosci..

[16] Yang, W. *et al.* Estimating the infection fatality risk of SARS-CoV-2 in New York City during the spring 2020 pandemic wave: a model-based analysis. *Lancet Infec Dis.*** 21**(2), 203–212 (2021). 10.1016/S1473-3099(20)30769-6 PMC7572090 33091374

[17] Verity R (2020). Estimates of the severity of coronavirus disease 2019: A model-based analysis. Lancet Infect. Dis..

[18] Green, M. S. *et al*. Sex differences in the case-fatality rates for COVID-19—A comparison of the age-related differences and consistency over seven countries. *PLoS ONE* **16**(4), e0250523 (2021) 10.1371/journal.pone.0250523 PMC8084161 33914806

[19] Buitrago-Garcia, D. C. *et al.* Occurrence and transmission potential of asymptomatic and presymptomatic SARS-CoV-2 infections: a living systematic review and meta-analysis. *PLoS Med* **17**(9), e1003346 (2020). 10.1371/journal.pmed.1003346 PMC7508369 32960881

[20] Gao, Z. *et al.* A systematic review of asymptomatic infections with COVID-19. *J. Microbiol. Immunol. Infect.* **54**(1), 12–16 (2021). 10.1016/j.jmii.2020.05.001 PMC7227597 32425996

[21] Yang J (2020). Prevalence of comorbidities and its effects in patients infected with SARS-CoV-2: A systematic review and meta-analysis. Int. J. Infect. Dis..

[22] Rodriguez-Morales AJ (2020). Clinical, laboratory and imaging features of COVID-19: A systematic review and meta-analysis. Travel Med. Infect. Dis..

[23] Sun P (2020). Clinical characteristics of hospitalized patients with SARS-CoV-2 infection: A single arm meta-analysis. J. Med. Virol..

[24] Lechien JR (2020). Olfactory and gustatory dysfunctions as a clinical presentation of mild-to-moderate forms of the coronavirus disease (COVID-19): A multicenter European study. Eur. Arch. Otorhinolaryngol..

[25] Passarelli PC, Lopez MA, Mastandrea Bonaviri GN, Garcia-Godoy F, Daddona A (2020). Taste and smell as chemosensory dysfunctions in COVID-19 infection. Am. J. Dent..

[26] Mao, L. *et al.* Neurological manifestations of hospitalized patients with COVID-19 in Wuhan, China: a retrospective case series study. *J. AMA Neurol*. **77**(6), 683–690 (2020). 10.1001/jamaneurol.2020.1127 PMC7149362 32275288

[27] Suresh Kumar VC (2020). Novelty in the gut: a systematic review and meta-analysis of the gastrointestinal manifestations of COVID-19. BMJ Open Gastroenterol..

[28] Parasa S (2020). Prevalence of gastrointestinal symptoms and fecal viral shedding in patients with coronavirus disease 2019: A systematic review and meta-analysis. JAMA Netw. Open.

[29] Cheung KS (2020). Gastrointestinal manifestations of SARS-CoV-2 infection and virus load in fecal samples from a Hong Kong Cohort: Systematic review and meta-analysis. Gastroenterology.

[30] Ministerio de Sanidad – Consejo Interterritorial del Sistema Nacional de Salud – Instituto de Salud Carlos III. Estudio ENE-COVID: informe final. Estudio nacional de sero-epidemiologia de la infección por SARS-CoV-2 en España. (Accessed 27 July 2020); https://www.mscbs.gob.es/ciudadanos/ene-covid/docs/ESTUDIO_ENE-COVID19_INFORME_FINAL.pdf (2020).

[31] Wynants L (2020). Prediction models for diagnosis and prognosis of covid-19 infection: Systematic review and critical appraisal. BMJ.

[32] Menni C (2020). Real-time tracking of self-reported symptoms to predict potential COVID-19. Nat. Med..

[33] Sudre, C. H. *et al.* Symptom clusters in Covid19: A potential clinical prediction tool from the COVID Symptom study app. *Sci Adv.* **7**(12), eabd4177 (2021) 10.1126/sciadv.abd4177 PMC7978420 33741586

[34] Bichara CDA (2021). Dynamics of anti-SARS-CoV-2 IgG antibodies post-COVID-19 in a Brazilian Amazon population. BMC Infect. Dis..

[35] Goldani LZ, Salort SG (2020). Infectious diseases and the COVID-19 scenario in Brazil. Braz. J. Infect. Dis..

