# Supplementary material for: High prevalence of symptoms among Brazilian subjects with antibodies against SARS-CoV-2
Source: Sci Rep. 2021 Jun 24;11:13279. doi: 10.1038/s41598-021-92775-y (PMC8225900; doi:10.1038/s41598-021-92775-y)
Supplement: Supplementary file 1 — Supplementary Information. [file 41598_2021_92775_MOESM1_ESM.docx]

Title:

High prevalence of symptoms among Brazilian subjects with antibodies against SARS-CoV-2

Authors:

Ana MB Menezes [anamene.epi@gmail.com](about:blank)  0000-0002-2996-9427

Cesar G Victora [cvictora@gmail.com](about:blank)   0000-0002-2465-2180

Fernando P Hartwig [fernandophartwig@gmail.com](about:blank) 0000-0003-3729-0710

Mariângela F Silveira [mariangelafreitassilveira@gmail.com](about:blank) [0000-0002-2861-7139](about:blank)

Bernardo L Horta [blhorta@gmail.com](about:blank)   0000-0001-9843-412X

Aluísio JD Barros [abarros.epi@gmail.com](about:blank)  0000-0002-2022-8729

Marilia A Mesenburg [mariliaepi@gmail.com](about:blank)  0000-0001-9598-4193

Fernando C Wehrmeister [fcwehrmeister@gmail.com](about:blank) 0000-0001-7137-1747

Lúcia C Pellanda [pellanda@ufcspa.edu.br](about:blank) [0000-0002-4593-3416](about:blank)

Odir A Dellagostin [odirad@gmail.com](about:blank)  0000-0003-2803-4088

Cláudio J Struchiner [claustru@gmail.com](about:blank)    0000-0003-2114-847X

Marcelo N Burattini [mnburatt@gmail.com](about:blank)  0000-0002-5407-6890

Fernando C Barros [fcbarros.epi@gmail.com](about:blank)    0000-0001-5973-1746

Pedro C Hallal [prchallal@gmail.com](about:blank) [0000-0003-1470-6461](about:blank)

Supplementary Table 1. Sensitivity and specificity of each symptom for identifying individuals with antibodies against SARS-CoV-2. The EPICOVID19 study, third wave.

| Symptom | Sensitivity | 95% CI | Specificity | 95% CI |
| --- | --- | --- | --- | --- |
| Headaches | 58.0 | 54.5 - 61.3 | 64.5 | 64.0 - 65.1 |
| Changes in smell or taste | 56.5 | 53.1 - 59.9 | 90.9 | 90.6 - 91.2 |
| Fever | 52.1 | 48.6 - 55.5 | 87.8 | 87.4 - 88.1 |
| Cough | 47.7 | 44.3 - 51.1 | 77.8 | 77.3 - 78.3 |
| Body aches | 44.1 | 40.7 - 47.5 | 84.3 | 83.9 - 84.7 |
| Sore throat | 33.8 | 30.6 - 37.1 | 83.4 | 83.0 - 83.8 |
| Diarrhea | 25.6 | 22.7 - 28.6 | 88.3 | 88.0 - 88.7 |
| Difficulty breathing | 23.1 | 20.3 - 26.1 | 90.6 | 90.3 - 90.9 |
| Shivering | 20.5 | 17.8 - 23.4 | 93.9 | 93.6 - 94.1 |
| Palpitation | 20.0 | 17.4 - 22.9 | 89.5 | 89.1 - 89.8 |
| Vomiting | 9.5 | 7.7 - 11.7 | 96.0 | 95.8 - 96.2 |

Supplementary Figure 1. Scatter diagram between prevalence of asymptomatic individuals with antibodies and seroprevalence for SARS-CoV-2, among the municipalities included in the study. The EPICOVID19 study, third wave.
